# A hybrid framework for glaucoma detection through federated machine learning and deep learning models

**DOI:** 10.1186/s12911-024-02518-y

**Published:** 2024-05-02

**Authors:** Abeer Aljohani, Rua Y. Aburasain

**Affiliations:** 1 Department of Computer Science , Applied College, Taibah University, Medina, 42353 Kingdom of Saudi Arabia; 2 Department of Computer Science, College of Engineering and Computer Science, Jazan University, Jazan, 45142 Kingdom of Saudi Arabia

**Keywords:** Machine learning, Deep learning, Convolutional neural network, Image processing and classification, Feature extraction, Glaucoma eye disease

## Abstract

**Background:**

Glaucoma, the second leading cause of global blindness, demands timely detection due to its asymptomatic progression. This paper introduces an advanced computerized system, integrates Machine Learning (ML), convolutional neural networks (CNNs), and image processing for accurate glaucoma detection using medical imaging data, surpassing prior research efforts.

**Method:**

Developing a hybrid glaucoma detection framework using CNNs (ResNet50, VGG-16) and Random Forest. Models analyze pre-processed retinal images independently, and post-processing rules combine predictions for an overall glaucoma impact assessment.

**Result:**

The hybrid framework achieves a significant 95.41% accuracy, with precision and recall at 99.37% and 88.37%, respectively. The F1 score, balancing precision and recall, reaches a commendable 93.52%. These results highlight the robustness and effectiveness of the hybrid framework in accurate glaucoma diagnosis.

**Conclusion:**

In summary, our research presents an innovative hybrid framework combining CNNs and traditional ML models for glaucoma detection. Using ResNet50, VGG-16, and Random Forest in an ensemble approach yields remarkable accuracy, precision, recall, and F1 score. These results showcase the methodology’s potential to enhance glaucoma diagnosis, emphasizing its promising role in early detection and preventing irreversible vision loss. The integration of ML and DNNs in medical imaging analysis suggests a valuable path for future advancements in ophthalmic healthcare.

**Supplementary Information:**

The online version contains supplementary material available at 10.1186/s12911-024-02518-y.

## Introduction

The human body has five senses: touch, hearing, sight, smell, and taste, but the sense of sight is used the most. Processing visual information involves a considerable portion of the brain [1]. Numerous diseases affect vision, like glaucoma, diabetic retinopathy, cataracts, amblyopia, refractive errors, and age-related macular degeneration. Among these diseases, glaucoma is the second most frequent reason for blindness worldwide [2, 3]. It can result in permanent vision loss within a few years and worsen over time.

**Fig. 1:**
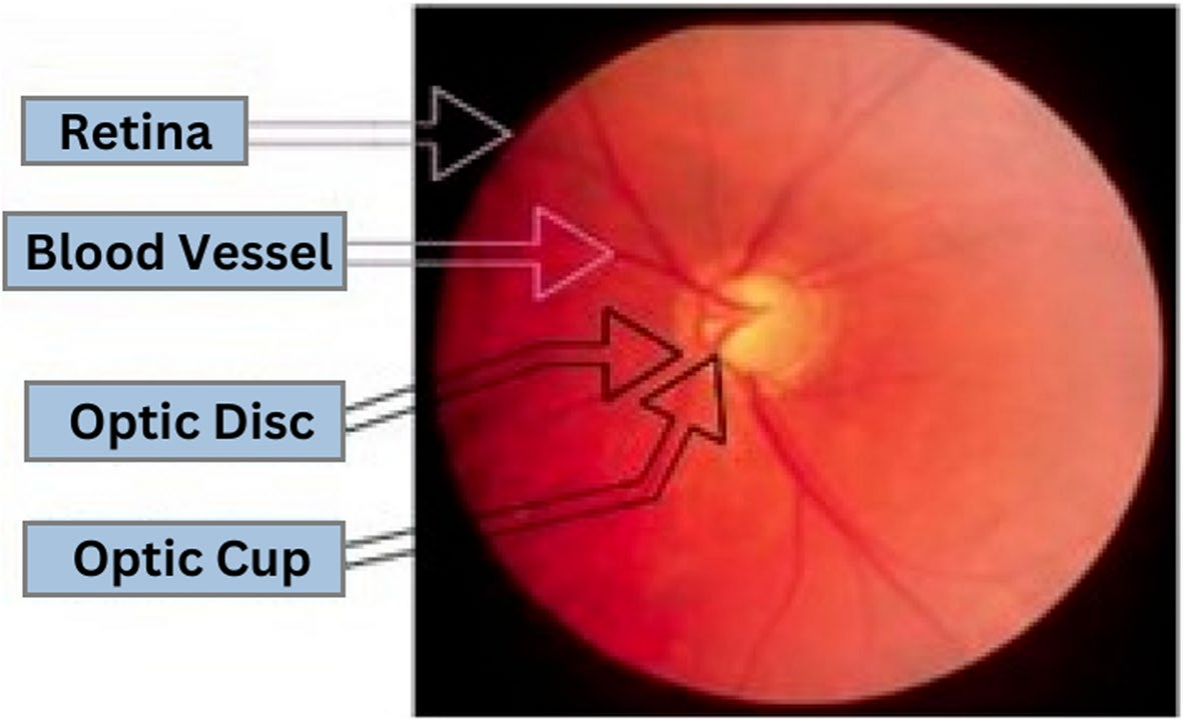
Labeled retinal fundus image of a glaucoma eye [4]

**Fig. 2:**
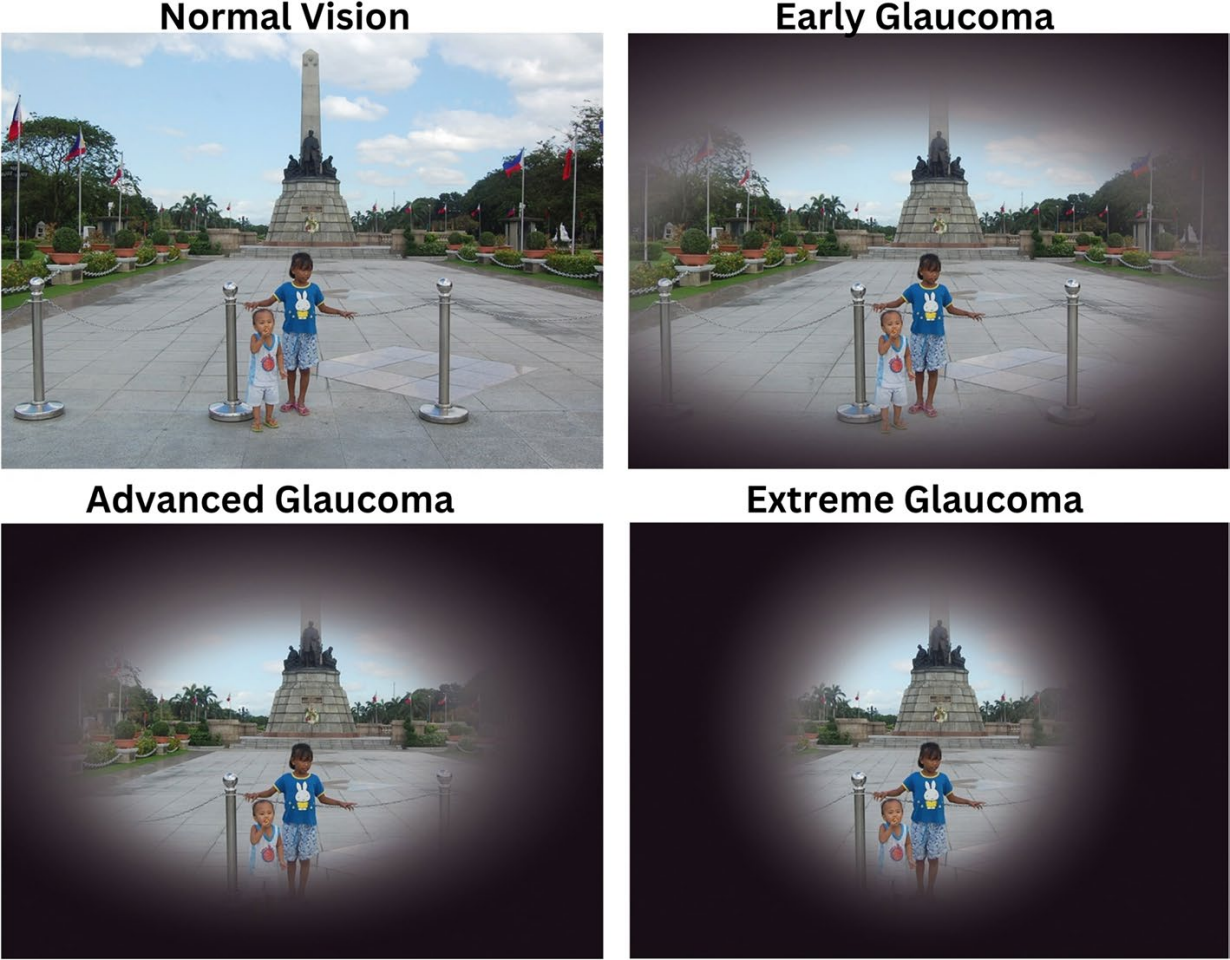
Normal Vision vs. Early Glaucoma vs. Advanced Glaucoma vs. Extreme Glaucoma [7]

Figure 1 depicts the retinal aspect of the eye, which has a glaucoma effect. The distance among the optic cup and the optic disc assists in recognizing glaucoma disease. The nerve fibers transmit messages from the eyes to the brain to form visual images, which could be harmed by the elevated intraocular pressure in the eyes [2, 4]. They are essential to the human eye’s capacity to see. Each optic nerve is made up of millions of nerve fibers. Damage to the optic nerve can cause visual loss in one or both eyes [5, 6]. The visual contrast between a normal eye and one with stages of glaucoma is depicted in Fig. 2.

According to existing literature, the World Health Organization projects that approximately 79 million to 112 million individuals may be affected by glaucoma by the years 2020 and 2040, respectively [1, 2, 4, 6, 8]. As per this analysis, it is imperative to diagnose glaucoma early or provide timely treatment. Machine Learning techniques, such as those powered by Artificial Intelligence, offer distinct advantages for detection and diagnosis, primarily through their capability to automate tasks efficiently, potentially improving early identification and intervention.

Artificial Intelligence (AI) is a vast field of computer science appurtenant to creating intelligent computers capable of doing activities that generally require human intelligence. Artificial Intelligence includes Machine Learning as a subset and a technique for data analysis that automates the development of analytical models and predicts a result based on data, spotting patterns and making judgments with corpuscle to no human involvement. Machine Learning (ML) has a subfield called Deep Learning (DL) that focuses on Artificial Neural Networks, a type of algorithm inspired by the design and operation of the brain [9] that handles structured and unstructured data types. Supervised Machine Learning requires human interruption to provide the features of the object to the model. In contrast, Deep Learning automatically learns and develops the features with substantially less manual interruption and produces high-order characteristics. Deep Learning provides a unique solution to solving many complicated, highly nonlinear problems [10]. The authors use Deep Learning to train the retinal fundus images to detect glaucoma for a desirable outcome, as Deep Learning has attracted massive research in healthcare. Convolutional Neural Network (CNN) is a form of Deep Learning extensively employed for the recognition and classification of images and objects. In this paper, classification has been done using ensemble ML and CNN models. There are three models used to achieve quality consequences. Random Forest, ResNet50, and VGG-16 models have been employed to address the issue. In a Random Forest, input is given as texture features [2, 11] which are extracted from retinal fundus images using the Gray-Level Co-Occurrence Matrix (GLCM) method. A framework has been proposed to detect whether the given input retinal fundus image belongs to glaucoma or normal.

### Motivation and objectives

A significant global health issue, glaucoma causes roughly 6.7 million people to become legally blind each year and is the subsequent largest cause of blindness globally [1, 2, 4, 6, 8, 12]. The primary motivation behind the research is to detect glaucoma-affected eyes, as this research is vital for human health. Earlier, less research has been done on glaucoma fundus detection in the healthcare industry, but as time went on, it became more significant to do research in this area. However, it is noted in the literature that an experiment had been conducted on a smaller number of images, so it is essential to experiment on a more significant number of images (refer to Table 2) for a high-quality result. Compared to other works of literature, the authors have employed a significant quantity of images. In literature, the work has been done either by using ML or CNN models, but in the proposed work, the authors have done work by assembling ML and CNN models in which ML uses texture data, and CNN uses image data. Affiliating the texture and image datasets for final classification has been crucial. The authors’ main objective is to see whether the given input retinal fundus image belongs to glaucoma or normal using ML and CNN models with post-processing rules.

### Background study

In [1], the researcher devised a Machine Learning-centered strategy for diagnosing glaucoma in individuals afflicted with the condition, utilizing three-dimensional Optical Coherence Tomography (OCT) data and color fundus images. The study incorporated 208 instances of glaucomatous and 148 healthy OCT data, resulting in an impressive accuracy of 96.3%. The analysis involved mapping thickness and deviation using a macular Ganglion Cell Complex (GCC), and a Convolutional Neural Network (CNN) was implemented through the application of transfer learning principles. In [4], the authors created a framework for detecting glaucoma using a CNN. To improve the local contrast, a framework applies the Contrast Limited Adaptive Histogram Equalization (CLAHE) as a preprocessing step. The optic cup and disc masks are segmented using EfficientNet [13] and U-Net [13], two segmentation models. The Cup-to-Disc Ratio (CDR) ratio, which is calculated from the segmented optic cup and disc, is the foundation of the framework that determines whether or not the input picture is glaucoma-infected. In the context of ocular health, a normal eye typically exhibits a Cup-to-Disc Ratio (CDR) value of 0.5 or less. Conversely, an eye affected by glaucoma is characterized by a CDR value exceeding 0.5. The application of benchmark datasets, specifically DRISHTI-GS1 and RIM-ONE, resulted in an accuracy rate of 91%. In [14], the authors developed a system that classifies glaucoma and non-glaucoma retinal fundus images known as Glaucoma-Deep and tested on 1200 images acquired from publicly and privately available datasets, namely DRIONS-DB, sjchoi86-HRF, HRF-dataset, PRV-Glaucoma. Glaucoma-Deep has achieved 99% accuracy using CNN and Deep-Belief Network (DBN).

In [15], the authors developed a system to classify fundus images into glaucoma and healthy images, which has been done using combined texture features and morphology optic nerve head and achieves 88.3% accuracy. They used the DRISHTI-GS dataset that provides 101 images consisting of 31 healthy images and 70 glaucoma images. To maintain a balanced class, the author considered only 60 images from that, of which 30 belongs to glaucoma images and other for healthy images. The Classification has been done using SVM and k-NN.

In [16], The authors developed a system utilizing convolutional neural networks (CNNs) to detect early-stage glaucoma by analyzing fundus images from datasets like ORIGA, STARE, and REFUGE. By employing pre-trained models such as ResNet50 and InceptionV3, the methodology aims to enhance medical diagnostic accuracy and efficiency. This approach establishes a reliable glaucoma diagnostic system, enabling accurate mass screenings and aiding ophthalmologists in early diagnosis, thereby improving patient outcomes.

In [17], The authors developed a system utilizing artificial intelligence algorithms for glaucoma diagnosis. This system explores glaucoma types, traditional diagnosis methods, and global disease epidemiology. It discusses AI’s potential in aiding early glaucoma detection and highlights progress in glaucoma classification algorithms. Challenges like database limitations and labeling inaccuracies are addressed, emphasizing the need for improved data diversity and standardization. Despite advancements, integrating AI into clinical practice remains limited, requiring further research for enhanced clinical utility.

## Methods

To achieve the objectives mentioned above, the authors use image processing techniques to extract texture features from the retinal fundus images and train the ML and CNN models using those images, to obtain quality consequences. In this approach, we introduce innovation through a comprehensive amalgamation of diverse texture features, the seamless integration of cutting-edge machine learning techniques, the careful curation and fine-tuning of CNN architectures, and an in-depth exploration of extensive and diverse datasets. These refinements collectively serve to surpass the limitations encountered in previous methodologies, profoundly enhancing the accuracy and robustness of glaucoma diagnosis. Ultimately, this advancement significantly bolsters both patient care and the progress of medical research in this field.

### Texture features

Features are necessary to obtain quality outcomes for complex problem statements in machine learning. Every time, the whole image may not require training the model, but it requires a needful area from an image according to the problem statement, which can be considered a feature that can enhance the final outcome. General image features, including texture, pattern, shape, color, edges, corners, region of interest, etc. The authors extracted texture features from retinal fundus images as the texture increased the texture description capability while simultaneously reducing the feature parameters [18]. There are methods for extracting texture features from the images [19–24], such as Gray-Level Co-Occurrence Matrix (GLCM) [2], GABOR filter [2, 24], Gray-Level Run-Length Matrix(GLRM) [25], histograms of gradient magnitudes, local energy patterns, etc. The authors have used the GLCM feature extraction methods in this research work and given it as an input to the Random Forest algorithm. The GLCM is a statistical method for analyzing texture that considers how pixels interact in space from various angles and distances. The GLCM values are easy to calculate and store since they are grayscale pixel values [26]. The features extracted with GLCM are [2, 27]. The choice of the GLCM method for texture feature extraction in this study is based on its ability to effectively capture intricate patterns, spatial relationships, and pixel interactions across various angles and distances. This method is favored for texture analysis because of its efficient feature calculation and the availability of rich texture descriptors. Its versatility proves valuable in image processing and machine learning, especially when dealing with extensive datasets, enabling the accurate differentiation of various textures.

The general equation to compute the GLCM is:

1 $$C\left(i,j,d\right)=\sum m=1 M\sum n=1 N\delta \left(I\left(m,n\right)=i\right)\delta \left(I\left(m+d,n\right)=j\right),$$

C(i, j,d) is the GLCM at offset d for pixel values i and j.

M and N are the dimensions of the image.

I(m, n) represents the pixel value at location (m, n).

δ(⋅) is the delta function that returns 1 if the condition is true and 0 otherwise.

### Machine learning

Machine Learning covers how to design machines that automatically better themselves through experience. The intersection of computer science and statistics, serving as the cornerstone for Artificial Intelligence and data science, positions it as one of the swiftly advancing technical domains in contemporary times [28]. Machine Learning algorithms create a model using training data to make predictions or judgments without being expressly coded. Machine learning techniques, as highlighted in studies such as [28], are employed to address challenges related to classification and regression. Some of these techniques include Random Forest [29], Linear Regression [30], Logistic Regression [31], Decision Tree [32], SVM [33], Naive Bayes [34], KNN Algorithm [29], and K-Means [29], among others. The authors conducted experiments incorporating texture features with various machine learning algorithms, including Random Forest, SVM, Naive Bayes, and Decision Tree, resulting in accuracy scores of 89.79%, 78.89%, 82.35%, and 84.56%, respectively. Based on the experiments and accuracy, the authors used Random Forest as an ML model for further research experiments. The Random Forest classifier is constructed from individual decision trees. These trees are created using a bootstrap sample of the data and a randomly selected set of features [29]. The process of tree building involves both bagging and random variable selection. After the forest is established, test samples pass through each tree, and the trees collectively predict the class. The error rate of the Random Forest is influenced by the strength of each tree and the correlation between any two trees. Additionally, it can be utilized to naturally rank the importance of variables in classification tasks. The authors have used the Random Forest algorithm to train the texture features extracted from retinal fundus images.

### CNN

Convolutional Neural Network (CNNs) are a form of Deep Neural Network. It adopts a distinctive method termed “convolution.” Convolution is a mathematical operation that combines two functions to generate a third function, illustrating how the shape of one function is altered by the other [35]. Since CNN does not require an individual handcrafted feature extraction approach, it is utilized explicitly for image reorganization, feature extraction, object detection, and image classification [36]. For image categorization and other objective perspectives that produce high-quality results with a high success rate, CNN has a variety of models available [36]. Various CNN models are LeNet [20, 37], AlexNet [27, 38], ResNet50 [13, 39], VGG-16 [4, 13], GoogleNet [40, 41], and MobileNet [13, 42, 43]. ResNet50 and VGG-16 have been adapted based on literature [4] to categorize the input retinal fundus images as either normal or glaucoma-related.

#### ResNet50

ResNet50 is a residual network having 50 layers and has been used in areas such as detection, segmentation, and identification. ResNet predicts the requisite delta to achieve the final prediction from one layer to the next. ResNet50 solves vanishing gradient issues [39], in which a deep multilayer feed-forward or recurrent neural network is unable to transport useful gradient information from the model’s output end to the layers near its input end.

ResNet50 was designed to address the vanishing gradient problem in deep neural networks by introducing skip connections, which allow information to flow more easily between distant layers. This innovation enables the training of very deep networks and has contributed to its excellent performance in computer vision tasks. It can learn an identity function, which enhances top-layer performance. There are five phases in all, each featuring a convolutional layer. To create the output block formula, a feed-forward neural network with condensed connections or a bottleneck structure may be utilized [39, 44]. A convolution of size 7 × 7 and 64 different kernels of size two are provided as the input to the first layer. These layers are joined together by a three-time convolution layer and a max-pooling layer with a stride number of two.

**Fig. 3:**
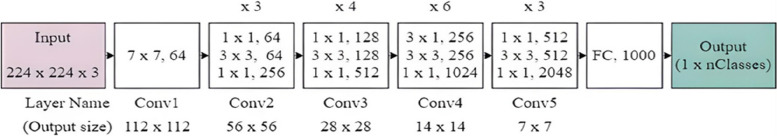
ConvNet architecture: ResNet50

Figure 3 depicts the Convolutional architecture of ResNet50. The deep residual learning framework comprises functions f(x) and y = x as identity mapping. The input to the block is appended as follows to the output block F(x):

2 $$\mathrm F(\mathrm x)\:=\:\mathrm f(\mathrm x)\:+\:\mathrm x,$$

ResNet50 uses a combination of convolutional layers to extract features, pooling layers to reduce spatial dimensions, residual connections to facilitate training of deep networks, and fully connected layers for classification. This architecture has been highly successful in image classification tasks and has paved the way for even deeper neural network architectures.

#### VGG-16

The VGG-16 network exhibits remarkable accuracies even when working with limited image datasets due to its extensive training. This network underwent training using the ImageNet database. It consists of two components and is a conventional neural network with 16 layers. The top two layers, consisting of 64 channels with 33 filter sizes, receive a retinal fundus image before sending it to the bottom two tiers. The max pool layer is followed by a max pool layer of stride (2, 2), two layers of 33 filters, two layers of convolution, and two levels of stride. The subsequent sequence of layers consists of two sets of three convolution layers iterated twice, coupled with a subsequent max pool layer. Following each convolution layer, an additional padding of one pixel is introduced to conceal the spatial details within the retinal fundus image.

**Fig. 4:**
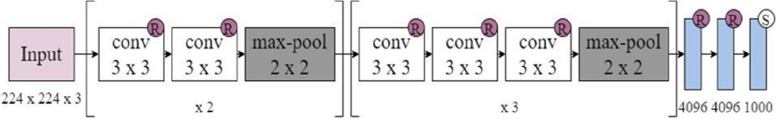
ConvNet architecture: VGGNet-16

Figure 4 depicts the convolutional architecture of VGG-16. A stack of convolution layers is followed by three connected layers and another pile of convolution layers. Each of the first and second levels has 4,096 channels, with the first layer having the most channels [39]. Within the VGG architecture, a dedicated memory space is allocated to store the feature vector of the top layer. Notably, the third layer, intricately linked to the SoftMax layer, encompasses 1000 channels.

## Proposed work

**Fig. 5:**
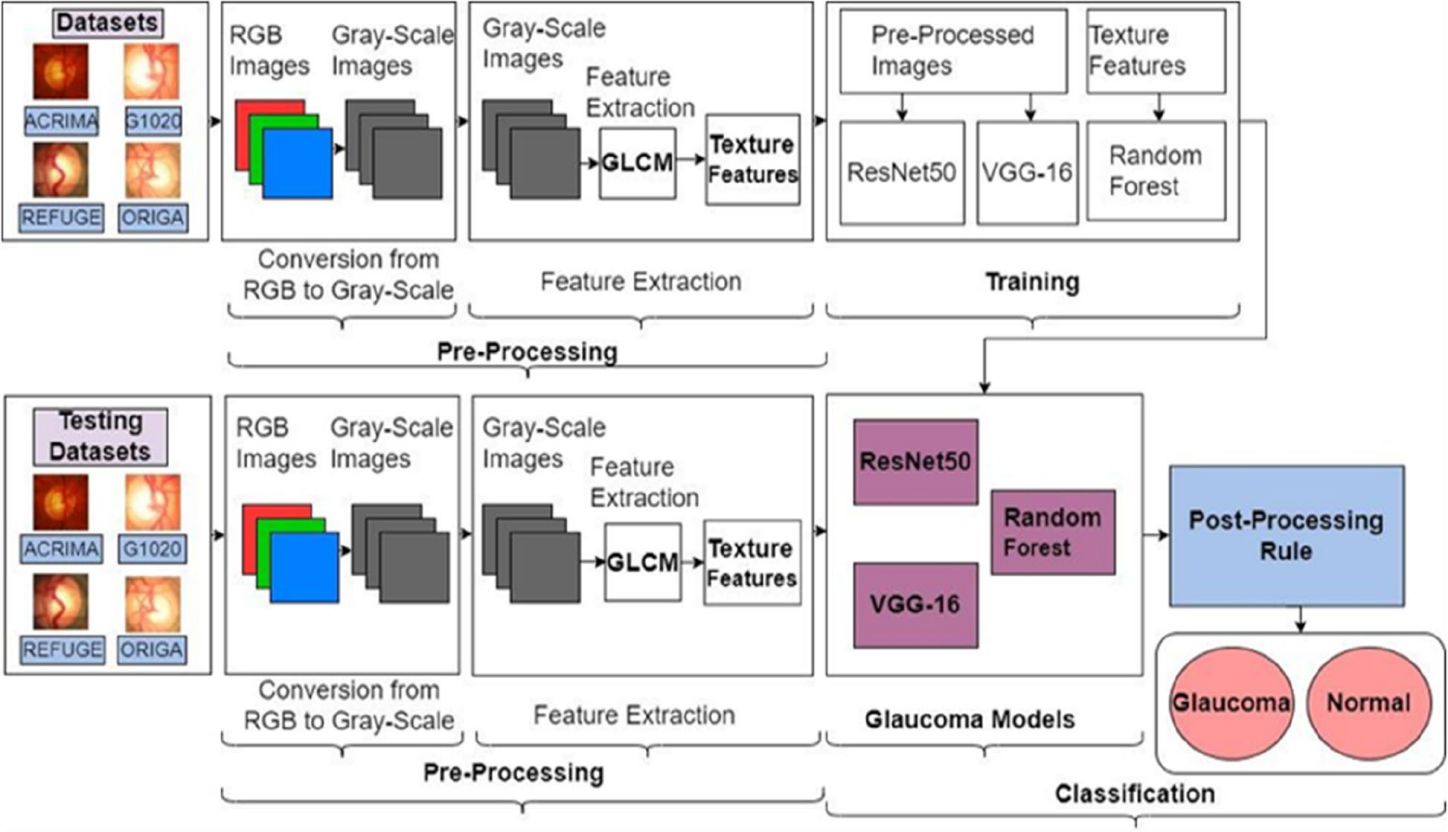
Proposed framework

The proposed work includes a glaucoma detection framework built with ensemble ML and CNN models. Framework made with three models, Random Forest, ResNet50, and VGG-16. The glaucoma detection framework preprocesses retinal digital images and generates texture features using GLCM, provided to the Random Forest algorithm and grayscale retinal digital images to ResNet50 and VGG-16. Once all three models have been saved, ensemble all together and, using the post-processing rule, classify the given input retinal fundus image as belonging to glaucoma or as normal as a final outcome. Figure 5 depicts the glaucoma detection framework to prevail on the problem statement, whether given an input retinal fundus image belongs to the glaucoma or normal category.

Based on the post-processing rule, the framework determines whether a particular input retinal fundus image is glaucoma-infected. If more than any two generated models predict glaucoma, then it will predict glaucoma as a final prediction; otherwise, it will predict as normal.

The proposed framework accommodates four major components. (1) Dataset Collection (2) Preprocessing (3) Training (4) Classification. Four benchmark datasets were used and integrated to achieve quality consequences with respect to the glaucoma detection framework to make the model more accurate. Preprocessing has been done in two phases: conversion from RGB images to grayscale images and feature extraction. Training has been done with 80% of the images in the dataset and has been performed on all three models, ResNet50, VGG-16, and Random Forest. Classification has been followed by post-processing rules with ensemble-saved trained models.

In the realm of predictive analytics, it is often encountered that sophisticated models may yield predict probabilities, yet they fall short in terms of accuracy when compared to the efficacy of post-processing rules. This observation underscores the significance of prioritizing rule-based approaches over model-driven ones, emphasizing that while advanced algorithms can provide probabilistic insights, the inherent power of well-crafted post-processing rules should not be underestimated.

### Dataset collection

In this research, the categorization of glaucoma has mainly been done using four standard datasets. These datasets are ACRIMA [5, 6, 42], G1020 [45], ORIGA [6, 42], and REFUGE [42]. These datasets include two categories of retinal fundus images, glaucoma and normal.

**Fig. 6:**
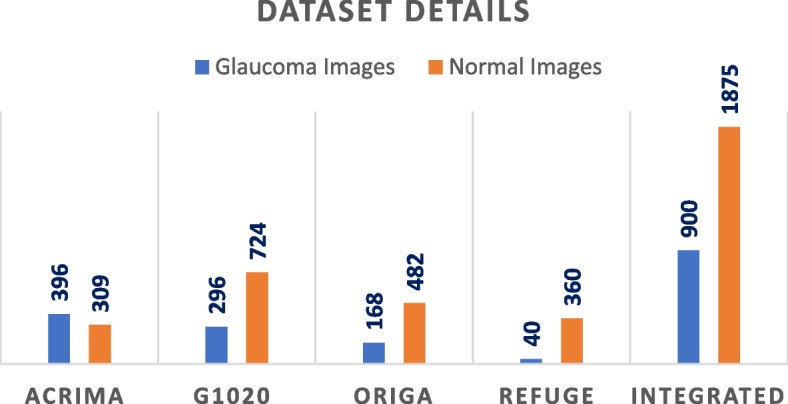
Dataset details

**Fig. 7:**
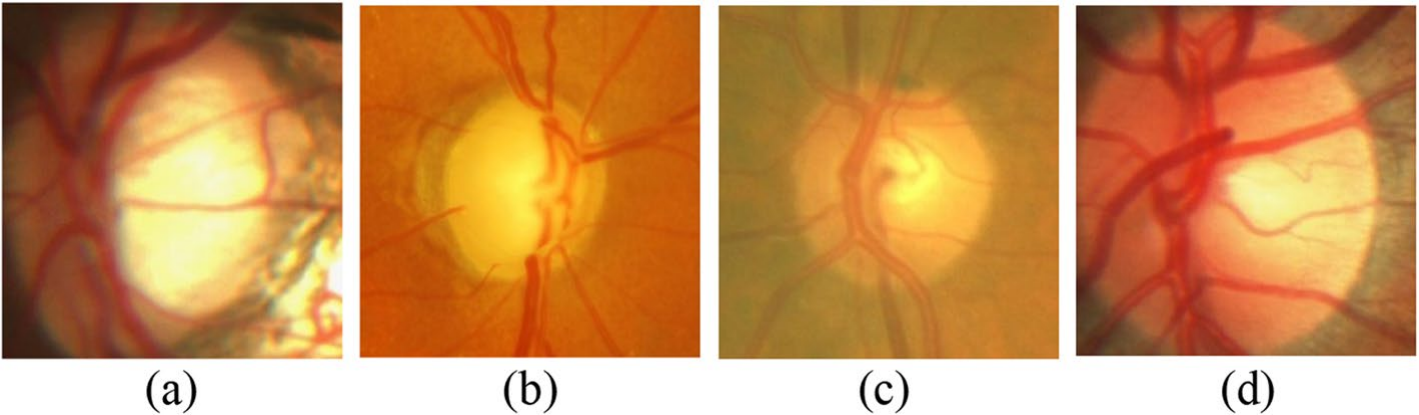
A few examples of the dataset’s contents. Consider that (**a**, **b**) indicates glaucoma, whereas (**c**, **d**) indicates non-glaucoma

Figure 6 shows the details of all the considered datasets for this research. All the datasets have been integrated for the classification of glaucoma. The dataset contains segmented retinal fundus RGB images in which the optic area is segmented for training and testing. The integrated dataset contains grayscale retinal fundus images converted from RGB retinal fundus images and used for training the models. Figure 7 illustrates a few instances from the dataset.

To enhance computation speed, each picture was scaled down to 224 × 224 × 3. To accelerate convergence and keep the model from becoming over- or underfit, images were shuffled in terms of position.

### Pre-processing

Data preprocessing is the first and most essential step in machine learning and deep learning. It organizes and cleans raw data to prepare for creating and training machine learning and CNN models. Cleaning the data is required to achieve a reasonable success rate. Preprocessing has been done in two phases for this research: (1) Conversion from RGB images to grayscale images (2) Feature Extraction.

#### Conversion from RGB to grayscale

**Fig. 8:**
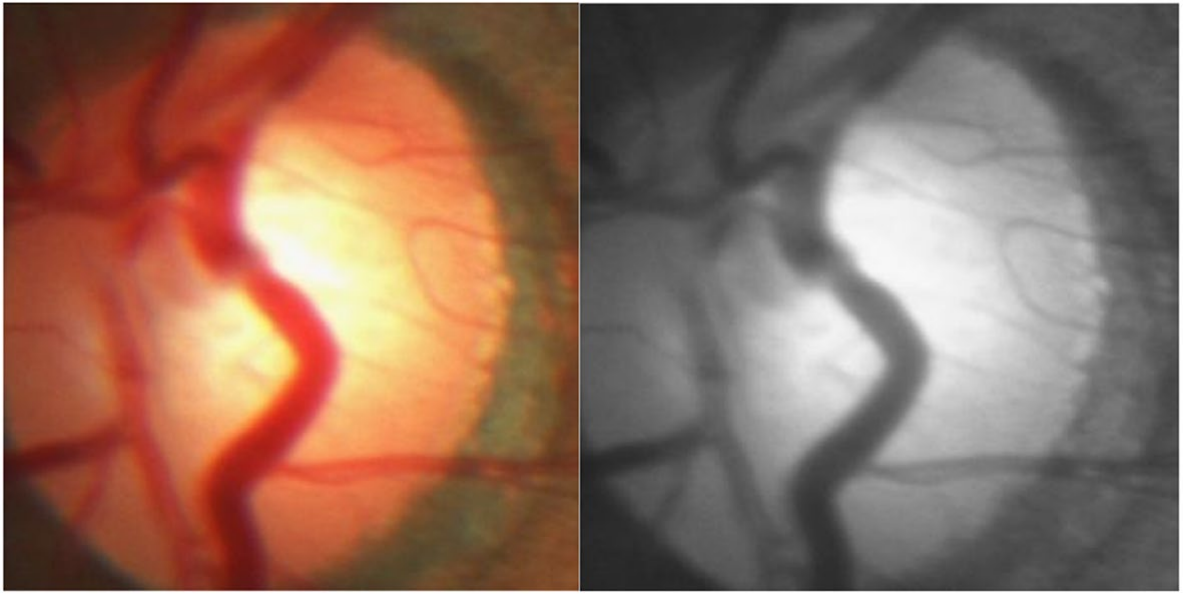
Conversion from RGB to grayscale

The authors converted retinal fundus images from RGB to grayscale. Grayscale retinal fundus images extract and identify texture features more accurately than RGB, reducing the noise and enhancing the results. The authors conducted experiments on RGB and grayscale retinal fundus images, and it was examined that efficient implicit features produced superior outcomes with grayscale images [46, 47]. The function rgb2gray() in python has been used to convert an RGB image to a grayscale image, as shown in Fig. 8.

The conversion from RGB to grayscale is typically performed using the following formula:

3 $$Gray=(w\_r*R+w\_g*G+{w}_{b}*B),$$

w_r(Red Weight):0.2989.

w_g(GreenWeight):0.5870.

w_b(Blue Weight):0.1140.

In the given expression, “Gray” denotes the grayscale pixel value, whereas R, G, and B symbolize the red, green, and blue pixel values in the RGB image, respectively. These weights are based on the perceived luminance of the color channels and are commonly employed in image processing for accurate grayscale conversion. The advantages of grayscale conversion in retinal fundus image analysis include improved texture feature extraction and noise reduction, which are essential for achieving superior results in various applications.

#### Feature extraction

Feature extraction is the most significant part of machine learning. The quantity of redundant data in the dataset is decreased with feature extraction. Reducing the feature space through techniques like GLCM on retinal fundus images is pivotal in machine learning. It not only eliminates redundancy but also offers vital advantages: it decreases learning parameters, enhancing computational efficiency, and preventing overfitting by implicitly applying regularization. This process results in simpler, faster, and more robust models, making feature extraction a crucial step in optimizing machine learning performance.

### Training

Glaucoma detection framework implemented using ML and CNN models. Training has been given to the ML model, Random Forest [29] and CNN models, ResNet50 [39, 44], and VGG-16 [39]. The ratio of splits for training and testing is 80:20. Texture features are given as input to the Random Forest. Retinal fundus grayscale images are provided as input to ResNet50 and VGG-16. The authors experimented on different hyperparameters to train the models, which is discussed in the result and discussion section.

## Results and discussion

The Glaucoma Detection Framework was evaluated using a personal laptop with an Intel core i5-9400 F CPU running at 2.90 GHz and 8 GB of RAM. The implementation used Keras [41, 48] and the TensorFlow [49] network in the Python Jupyter notebook [50], and the Python [51] language was also used to statistically calculate the outcomes. In the context of our glaucoma detection framework, accuracy is a crucial metric for evaluating the performance of our model. The accuracy formula used in our research is defined as:

$$\text{A}\text{c}\text{c}\text{u}\text{r}\text{a}\text{c}\text{y}=\frac{\left(\text{T}\text{P}+\text{T}\text{N}\right)}{\left(\text{T}\text{P}+\text{F}\text{P} +\text{T}\text{N}+\text{F}\text{N} \right)}$$

where:

- TP represents the number of true positive cases (correctly forecasted positive cases).
- TN represents the number of true negative cases (properly projected negative cases).
- FP represents the number of false positive cases (mistakenly forecasted positive cases).
- FN represents the number of false negative cases (mistakenly forecasted negative cases).

This formula is widely used in binary classification scenarios, where the goal is to distinguish between two classes, in our case, the presence or absence of glaucoma. The key components of the formula are:

- True Positive (TP): These are instances where the model correctly identifies positive cases, in our case, correctly forecasting the presence of glaucoma.
- True Negative (TN): These are instances where the model correctly identifies negative cases, accurately predicting the absence of glaucoma.
- False Positive (FP): These are instances where the model incorrectly predicts positive cases, indicating the presence of glaucoma when it is not actually present.
- False Negative (FN): These are instances where the model incorrectly predicts negative cases, failing to identify the presence of glaucoma when it is present.

The accuracy formula essentially quantifies the overall correctness of the model’s predictions by considering both positive and negative cases. It provides a comprehensive assessment of the model’s ability to correctly classify instances, making it a valuable metric in evaluating the performance of our glaucoma detection framework.

**Fig. 9:**
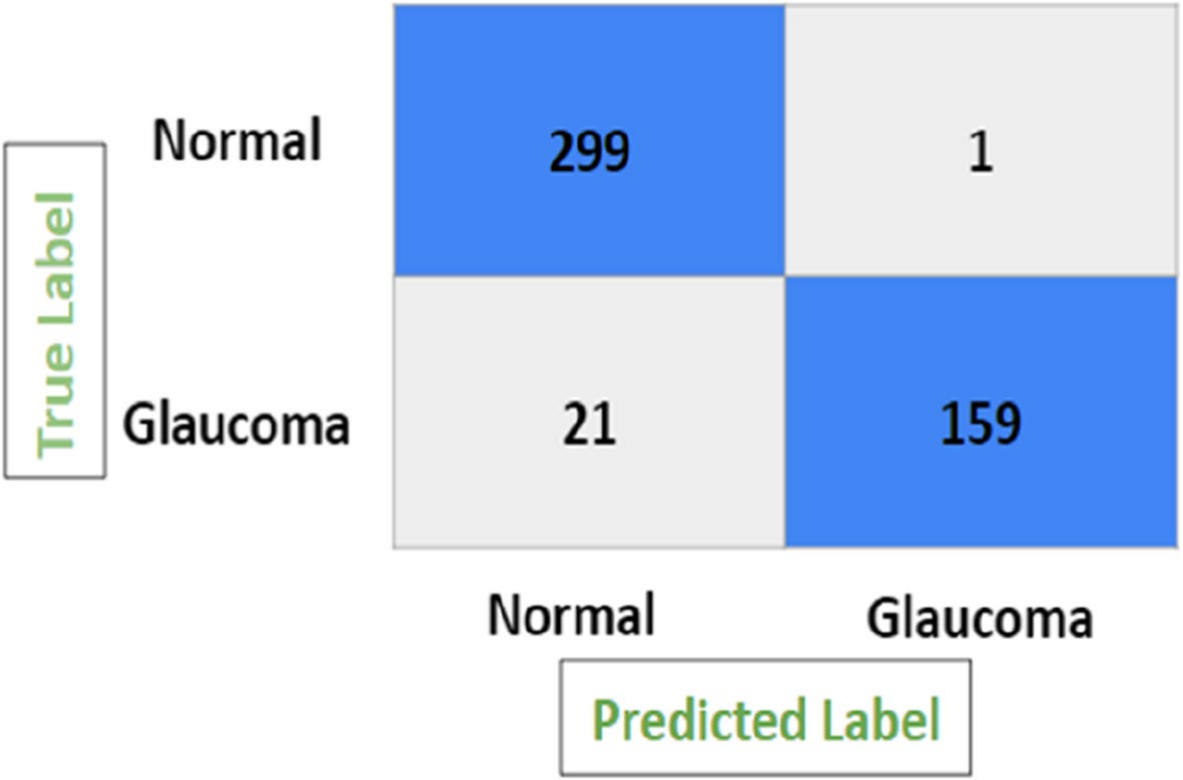
Confusion matrix of glaucoma detection framework

Figure 9 depicts the confusion matrix for the glaucoma classification, which shows the number of all correct and incorrect predictions. With a true positive rate of 88.33%, a true negative rate of 99.66%, a false discovery rate of 4.59%, and a positive predictive value of 95.41%, this approach has a high degree of accuracy. Authors have experimented on each model for hyperparameters described in the subsections below.

### Random forest

The Random Forest method mixes numerous decision trees and navigates complicated challenges to produce the final result [29]. Texture features are used as input to train the Random Forest, such as dissimilarity, correlation, homogeneity, contrast, ASM, and energy extracted using GLCM.

**Fig. 10:**
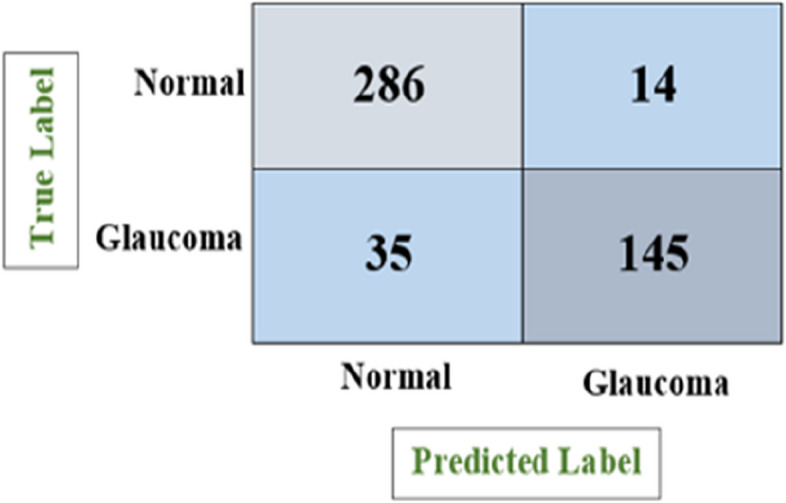
Confusion matrix of random forest

Figure 10 depicts the confusion matrix for the glaucoma classification using Random Forest model, which shows the number of all correct and incorrect predictions and achieved accuracy of 89.79%, precision of 91.19%, recall of 80.56% and F1-score of 85.59%.

### ResNet50

ResNet50 is the CNN model used for classification and regression problems. It consists of 50 layers. To train the ResNet50 model, Retinal grayscale fundus images have been used as input data in which specific parameters have been used, like the number of epochs, batch size, activation function, optimizer, and shuffle. There are 50 epochs with a batch size of 32; the activation function is as it doesn’t suffer from the vanishing gradient problem, the optimizer is Adam to speed up the time to classifying images [44], and the shuffle is false. All these parameters have been decided based on the literature review [39, 44]. For a more comprehensive understanding of hyperparameters, please refer to Appendix A.

**Fig. 11:**
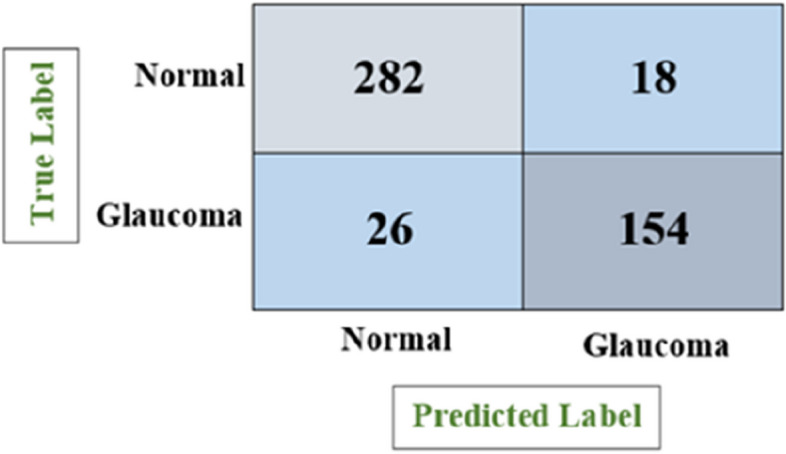
Confusion matrix of ResNet50

Figure 11 depicts the confusion matrix for the glaucoma classification using ResNet50 model, which shows the number of all correct and incorrect predictions and achieved accuracy of 90.83%, precision of 89.53%, recall of 85.55% and F1-score of 87.47%.

### VGG-16

VGG-16 is the CNN model used for classification and regression problems. It consists of 16 layers. To train the VGG16 model, Retinal grayscale fundus images have been used as input data in which specific parameters have been used, like the number of epochs, batch size, activation function, optimizer, and shuffle. There are 50 epochs with a batch size of 32; the activation function is as it doesn’t suffer from the vanishing gradient problem, the optimizer is Adam to speed up the time for classifying images, and the shuffle is false. All these parameters have been decided based on the literature review [39]. For a more comprehensive understanding of hyperparameters, please refer to Appendix B.

**Fig. 12:**
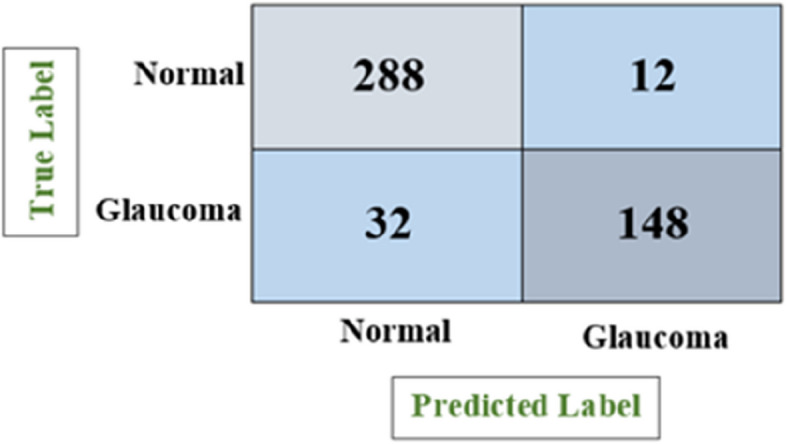
Confusion matrix of VGG-16

Figure 12 depicts the confusion matrix for the glaucoma classification using VGG-16 model, which shows the number of all correct and incorrect predictions and achieved accuracy of 90.83%, precision of 92.50%, recall of 82.22% and F1-score of 87.03%.

### Glaucoma detection framework

The authors have conducted experiments with the ML algorithm; Random Forest, in which texture features have been given as input data and got an accuracy of 89.79% and concluded that accuracy achieved by the ML algorithm is not sufficient for good prediction. Other experiments were conducted with CNN models to make the model more accurate in predicting genuine consequences. Still, as those models also did not give the expected accuracy, the authors introduce the glaucoma detection framework in which ML and CNN models have been ensembled to improve overall accuracy.

**Fig. 13:**
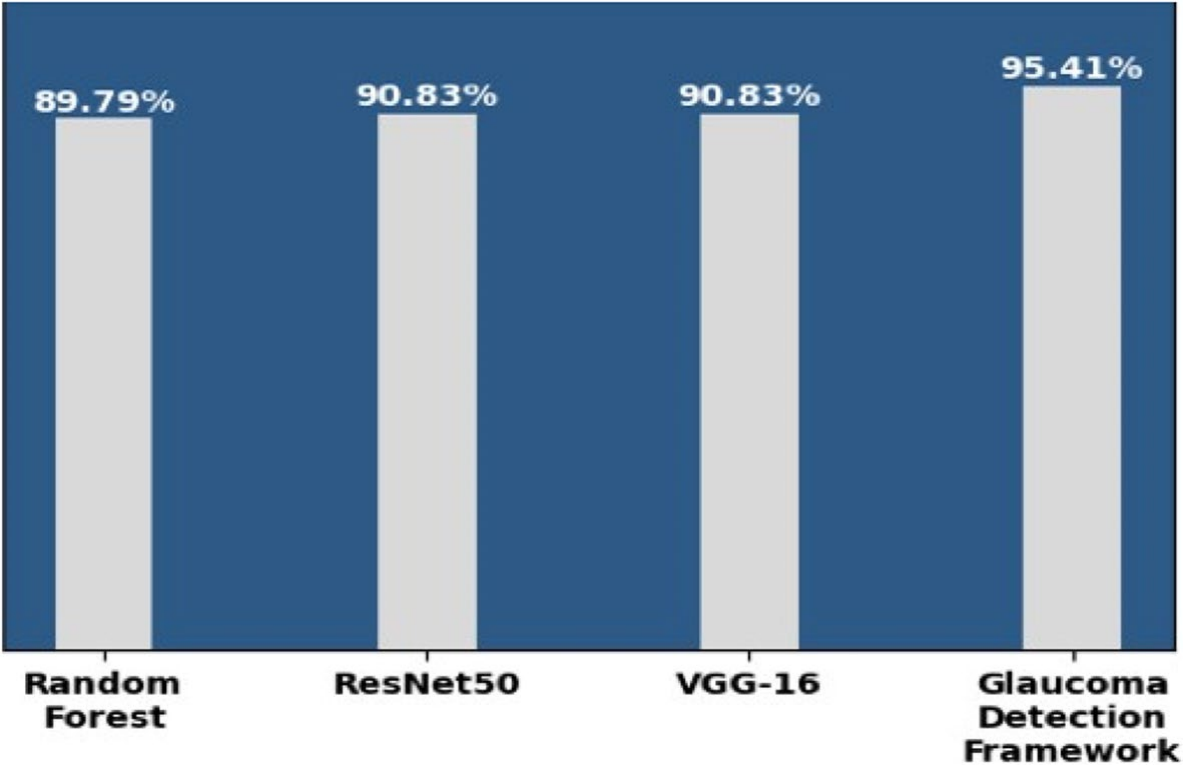
Models and accuracy

Figure 13 depicts the accuracy score of the ML model, CNN models, and the glaucoma detection framework.

Figure 13 shows that the glaucoma detection framework made from an ensemble of the ML and CNN models gives improved results compared to individual ones.

It demonstrates that the accuracy of the Random Forest, ResNet50, and VGG16 is, respectively, 89.79%, 90.83%, and 90.83%. In contrast, the accuracy of the proposed glaucoma detection framework is 95.41% using the integrated dataset and hybrid approach. Glaucoma detection framework performs on Random Forest, ResNet50, and VGG16 and predict result individually for similar input. Post-processing rule apply on all three model’s results and make a final classification which gives more precise outcome compare to individual model’s outcome. The post-processing rule is that the final prediction will be ‘glaucoma’ if at least two generated models independently indicate it as ‘glaucoma’; otherwise, it will be predicted as ‘normal’.

**Fig. 14:**
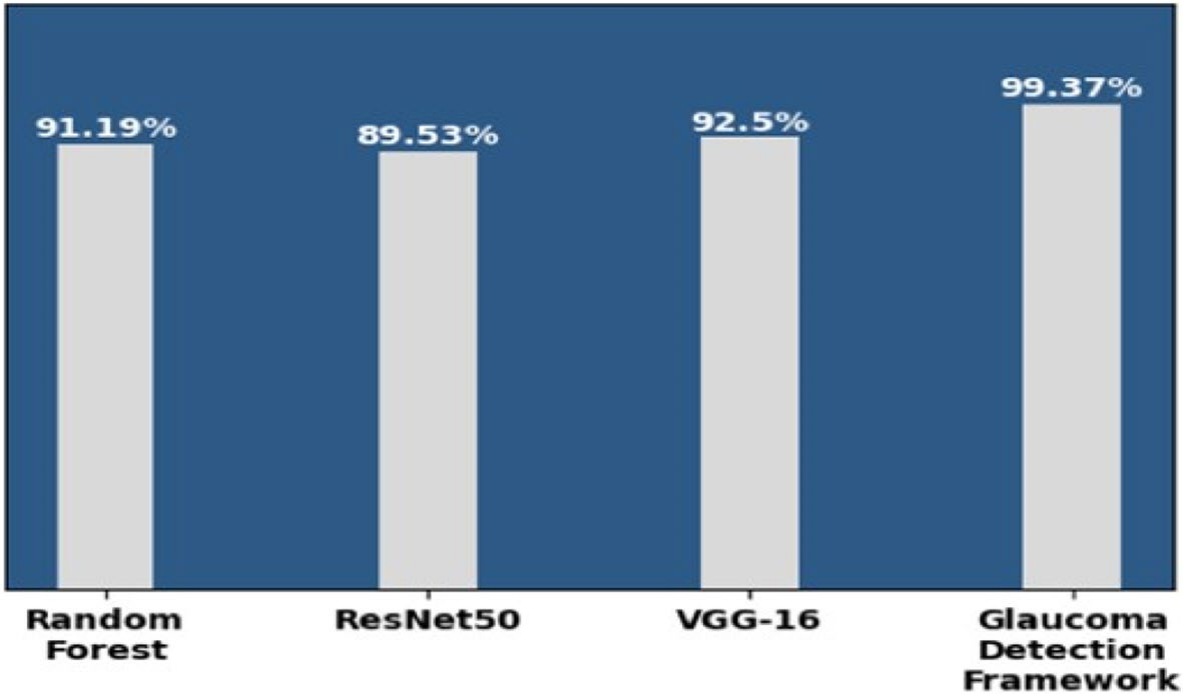
Models and precision

Figure 14 demonstrates that the precision of the Random Forest, ResNet50, and VGG16 is, respectively, 91.19%, 89.53%, and 92.50%. In contrast, the precision of the proposed glaucoma detection framework is 99.37% using the integrated dataset and hybrid approach.

**Fig. 15:**
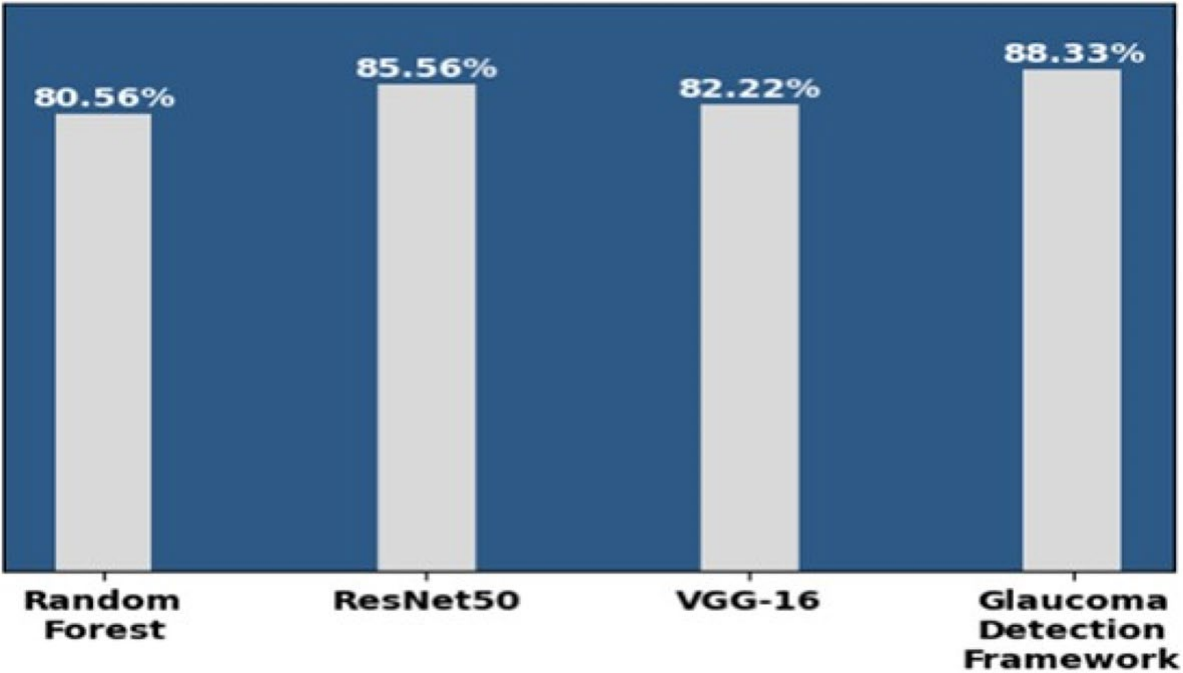
Models and recall

Figure 15 demonstrates that the recall of the Random Forest, ResNet50, and VGG16 is, respectively, 80.56%, 85.56%, and 82.22%. In contrast, the recall of the proposed glaucoma detection framework is 88.33% using the integrated dataset and hybrid approach.

**Fig. 16:**
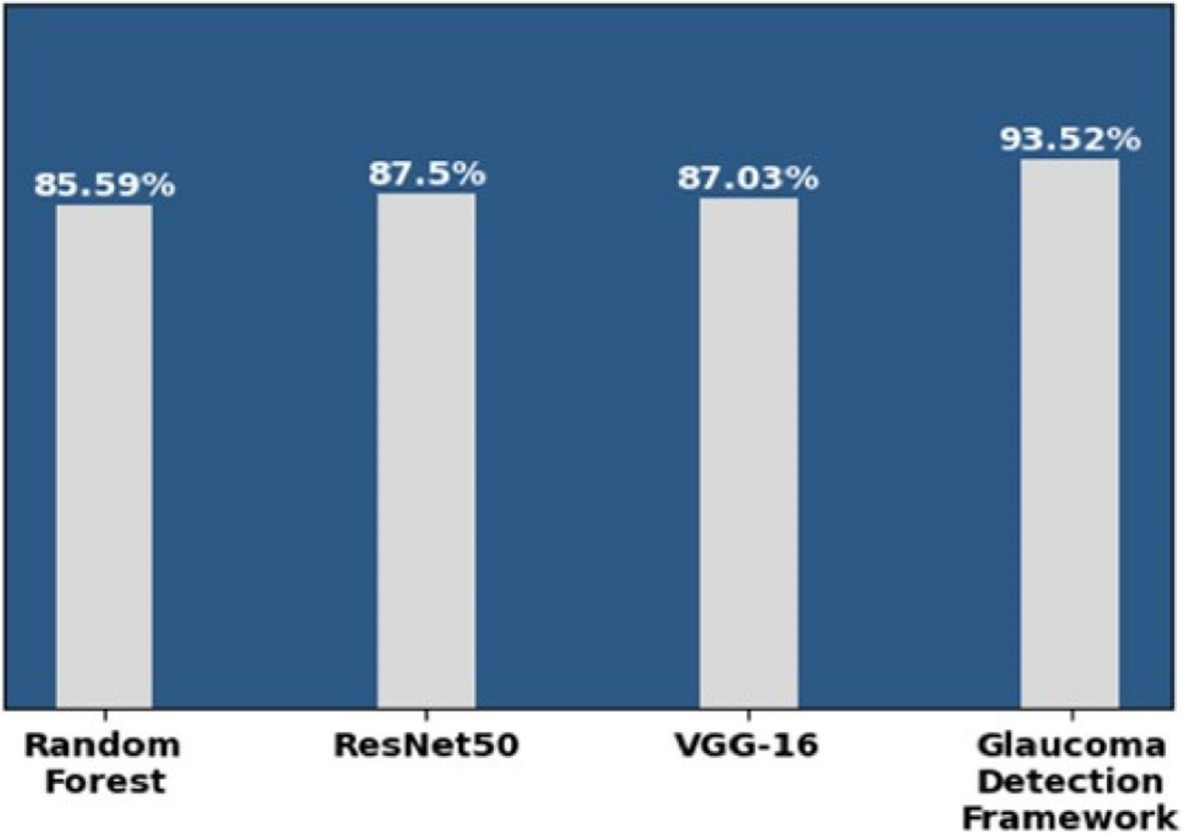
Models and F1-Score

Figure 16 demonstrates that the F1-Score of the Random Forest, ResNet50, and VGG16 is, respectively, 85.59%, 87.50%, and 87.03%. In contrast, the F1-Score of the proposed glaucoma detection framework is 93.52% using the integrated dataset and hybrid approach. The Table 1 below outlines the justifications for opting for post-processing rules.

**Table 1:** Post-processing rule justification

x/n Rule	No. of True Prediction based on Rule	Justification
1/3 to Predict Glaucoma Diagnosis	14	Too lenient, may yield many false positives.
2/3 to Predict Glaucoma Diagnosis	85	Strikes a balance b/w sensitivity and specificity. Captures a significant number of true cases (85).
3/3 to Predict Glaucoma Diagnosis	373	Very conservative, may miss many true cases.
2/3 + 3/3 to Predict Glaucoma Diagnosis (Post-processing Rule)	458	Combines the above rules, capturing even more true cases (458).

In above Table 1, the “x/n” notation signifies a predictive rule where “x” indicates the number of models predicting true for Glaucoma Diagnosis out of a total of “n” models. In the context of our study, “1,” “2,” and “3” correspond to the quantity of models out of the three available that predict true for Glaucoma Diagnosis. For instance, “2/3” implies that two out of the three models are predicting true for Glaucoma Diagnosis. Out of a total of 480 test cases, 458 of them resulted in accurate predictions.

The “2/3 or more models predicting Glaucoma” post-processing rule was adopted because it strikes a balance between comprehensiveness and accuracy. It captures a significant number of true Glaucoma cases while maintaining some level of stringency by requiring at least two out of three models to concur. This choice avoids the overly conservative nature of the “3/3 to predict Glaucoma Diagnosis” rule, which might miss many true cases, and the overly permissive “1/3 to predict Glaucoma Diagnosis” rule, which could introduce numerous false positives. Consequently, the “2/3 or more models predicting Glaucoma Diagnosis” rule optimally balances accurate Glaucoma detection and reduced false positives, as supported by the analysis of true predictions in the data.

### Comparison with cutting-edge techniques

In Table 2, the developed model’s classification performance is compared to that of several cutting-edge techniques. The authors chose current models based on DL and ML approaches to improve performance coherence and relevance. The comparison of the proposed model with other cutting-edge methods depicts in Table 2.

**Table 2:** Comparison of the proposed model with other cutting-edge approaches

Ref	Method	Dataset Name	Dataset (Images)	Accuracy (%)	Precision (%)	Recall (%)	F1-Score (%)
[29]	RF	REFUGE	400 Images	75.86	71.72	76.24	71.42
Proposed Work	RF	ACRIMA, G1020, ORIGA, REFUGE	2775 Images	95.41	99.37	88.33	93.52
	ResNet50						
	VGG16						

Table 2 makes it evident that the proposed work’s accuracy is higher when evaluated against the dataset and the performance of other cutting-edge methodologies [1]. used CNN with 356 trained images and achieves 96.3% accuracy but the proposed approach used ML and CNN with 2775 trained images and achieves 95.41% accuracy. From Table 2, it has been concluded that researchers used either only ML or CNN approach with less amount of images, but the proposed approach uses both approaches with the hybrid model concept, and more images compared to the literature and achieves 95.41% accuracy.

### Clinical significance

This study’s findings have significant implications for glaucoma detection using machine learning. It emphasizes the importance of combining traditional optic disc measurements with image texture-derived features in machine learning models to improve early and accurate glaucoma diagnosis. By incorporating texture-based information, these models can detect subtle signs of glaucoma that might be missed by conventional methods alone, enhancing diagnostic accuracy and reliability.

Furthermore, the research highlights that machine learning models integrating diverse feature sets, including structural and texture-based features, exhibit strong generalization capabilities. This is essential for clinical applications, as it ensures effective performance across different datasets and patient populations. Clinicians and researchers can use these insights to develop robust glaucoma detection models that can adapt to variations in imaging devices, patient demographics, and disease presentations. Ultimately, this research enables better early glaucoma detection, leading to improved patient outcomes and more effective disease management.

## Conclusion

Every year, millions of individuals worldwide are impacted by glaucoma, a retinal illness. It results in irreversible blindness if it is not caught in time. The main objective of this research is glaucoma detection and its classification. The authors proposed a glaucoma detection framework to distinguish between glaucoma eyes and normal eyes using the post-processing rule to achieve the above objective. The final prediction will be ‘glaucoma’ if at least two generated models independently indicate it as ‘glaucoma’; otherwise, it will be predicted as ‘normal.’ An integrated dataset was generated from four public datasets: ACRIMA, G1020, ORIGA, and REFUGE. Texture features and retinal fundus image has been used to develop a glaucoma detection framework. Texture features such as dissimilarity, correlation, homogeneity, contrast, ASM, and energy have been extracted using GLCM and given as input to the Random Forest. Retinal grayscale fundus images have been provided as input to the ResNet50 and VGG16. The glaucoma detection framework was meticulously developed through ensemble modeling, incorporating Random Forest, ResNet50, and VGG16. This comprehensive approach yielded impressive results: an accuracy of 95.41%, a precision rate of 99.37%, a recall rate of 88.33%, and an F1-score of 93.52%.

### Supplementary Information

**Supplementary Material 1.**

## Data Availability

The data used in this study were freely obtained from the Kaggle website and are available at https://www.kaggle.com/datasets/sshikamaru/glaucoma-detection, https://www.kaggle.com/datasets/hindsaud/datasets-higancnn-glaucoma-detection. Interested researchers can access the data by visiting the respective Kaggle website and following the provided guidelines for data retrieval.
