# Supplementary material for: A hybrid framework for glaucoma detection through federated machine learning and deep learning models
Source: BMC Med Inform Decis Mak. 2024 May 2;24:115. doi: 10.1186/s12911-024-02518-y (PMC11064392; doi:10.1186/s12911-024-02518-y)
Supplement: Supplementary file 1 — Supplementary Material 1. [file 12911_2024_2518_MOESM1_ESM.pdf]

Appendix A: ResNet50 Hyperparameter Tuning: A Comprehensive Approach

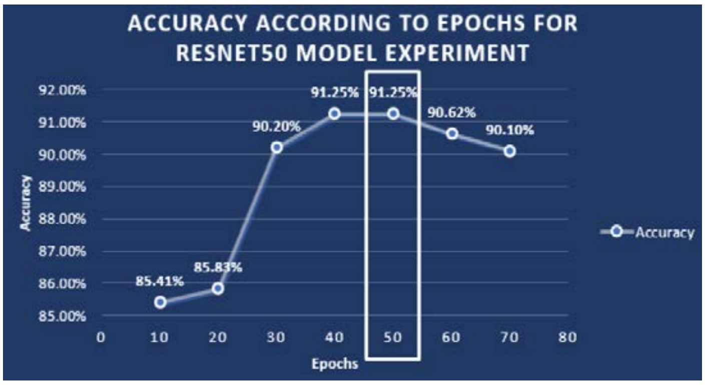

Fig. A.1 Accuracy according to epochs for the ResNet50 model experiment

The author conducted experiments on numerous epochs for the ResNet50 model and analyzed the accuracy accordingly, as shown in Fig. A.1. The author concluded after analyzing the epochs' activities that accuracy is steady for the first 50 epochs, but after that, accuracy decreases. So, the ideal epoch number for the ResNet50 experiment is 50.

The author evaluated the accuracy, precision, recall, and F1-Score from epochs data for the proposed model's performance for each class, as shown in Table A.1.

Table A.1 Epoch results (after 10 epochs, in %): Precision, Recall, and F1-Score

**Epoch**	**Class**	**Precision**	**Recall**	**F1-Score**
10	Not Glaucoma	82.14	94.78	88.01
	Glaucoma	74.66	42.74	54.36
20	Not Glaucoma	80.20	93.76	86.45
	Glaucoma	79.20	50.63	61.77
30	Not Glaucoma	85.33	87.91	86.60
	Glaucoma	63.33	58.01	60.55
40	Not Glaucoma	83.79	90.93	87.22
	Glaucoma	67.00	51.14	58.00
**50**	**Not Glaucoma**	**82.13**	**91.39**	**86.51**
	**Glaucoma**	**75.83**	**57.59**	**65.46**
$\mu \pm \sigma$	Not Glaucoma	82.71 ± 1.73	91.75 ± 2.39	86.95 ± 0.59
	Glaucoma	72.00 ± 5.89	52.02 ± 5.58	60.02 ± 2.99

As illustrated in Table A.1, the effectiveness of the ResNet50 model for each class, as well as the accuracy, precision, recall, and F1-score from epochs data, were analyzed on a category basis. Table A.1 shows that the Hybrid model has the following results: accuracy of 72.00 ± 5.89–82.71 ± 1.73, recall of 52.02 ± 5.58–91.75 ± 2.39, and F1-Score of 60.02 ± 2.99–86.95 ± 0.59. The ResNet50 model has the maximum F1-Score in the "Not Glaucoma" class, indicating that it is receptive to such features.

Appendix B: VGG-16 Hyperparameter Tuning: A Comprehensive Approach

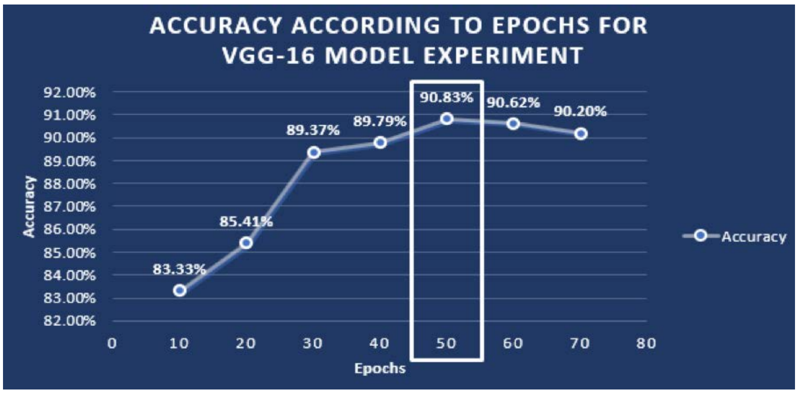

Fig B.1 Accuracy according to epochs for the VGG-16 model experiment

The author conducted experiments on numerous epochs for the VGG-16 model and analyzed the accuracy accordingly, as shown in Fig B.1. The author concluded after analyzing the epochs' activities that accuracy is steady for the first 50 epochs, but after that, accuracy decreases. So, the ideal epoch number for the VGG-16 experiment is 50.

The author evaluated the accuracy, precision, recall, and F1-Score from epochs data for the proposed model's performance for each class, as shown in Table B.1.

Table B.1 Epoch results (after 10 epochs, in %): Precision, Recall, and F1-Score

**Epoch**	**Class**	**Precision**	**Recall**	**F1-Score**
10	Not Glaucoma	82.03	86.53	84.22
	Glaucoma	55.85	47.32	51.24
20	Not Glaucoma	80.54	88.73	84.44
	Glaucoma	56.38	40.45	47.11
30	Not Glaucoma	82.99	79.12	81.01
	Glaucoma	48.64	54.96	51.61
40	Not Glaucoma	80.42	83.51	81.94
	Glaucoma	48.71	43.51	45.96
**50**	**Not Glaucoma**	**79.56**	**90.93**	**84.87**

	**Glaucoma**	**58.22**	**35.11**	**43.81**
μ ± σ	Not Glaucoma	81.10 ± 1.23	85.76 ± 4.13	83.29 ± 1.53
	Glaucoma	53.56 ± 4.06	44.27 ± 6.67	47.94 ± 3.03

As illustrated in Table B.1, the effectiveness of the VGG-16 model for each class, as well as the accuracy, precision, recall, and F1-score from epochs data, were analyzed on a category basis. Table B.1 shows that the Hybrid model has the following results: accuracy of 53.56 ± 4.06–81.10 ± 1.23, recall of 44.27 ± 6.67–85.76 ± 4.13, and F1-Score of 47.94 ± 3.03–83.29 ± 1.53. The VGG-16 model has the maximum F1-Score in the "Not Glaucoma" class, indicating that it is receptive to such features.
